# Dietary docosahexaenoic acid (DHA) as lysophosphatidylcholine, but not as free acid, enriches brain DHA and improves memory in adult mice

**DOI:** 10.1038/s41598-017-11766-0

**Published:** 2017-09-12

**Authors:** Dhavamani Sugasini, Riya Thomas, Poorna C. R. Yalagala, Leon M. Tai, Papasani V. Subbaiah

**Affiliations:** 1 Department of Medicine, University of Illinois at Chicago, Chicago, IL USA; 2 Department of Anatomy and Cell Biology, University of Illinois at Chicago, Chicago, IL USA; 3 Jesse Brown VA Medical Center, Chicago, IL USA

## Abstract

Docosahexaenoic acid (DHA) is uniquely concentrated in the brain, and is essential for its function, but must be mostly acquired from diet. Most of the current supplements of DHA, including fish oil and krill oil, do not significantly increase brain DHA, because they are hydrolyzed to free DHA and are absorbed as triacylglycerol, whereas the transporter at blood brain barrier is specific for phospholipid form of DHA. Here we show that oral administration of DHA to normal adult mice as lysophosphatidylcholine (LPC) (40 mg DHA/kg) for 30 days increased DHA content of the brain by >2-fold. In contrast, the same amount of free DHA did not increase brain DHA, but increased the DHA in adipose tissue and heart. Moreover, LPC-DHA treatment markedly improved the spatial learning and memory, as measured by Morris water maze test, whereas free DHA had no effect. The brain derived neurotrophic factor increased in all brain regions with LPC-DHA, but not with free DHA. These studies show that dietary LPC-DHA efficiently increases brain DHA content and improves brain function in adult mammals, thus providing a novel nutraceutical approach for the prevention and treatment of neurological diseases associated with DHA deficiency, such as Alzheimer’s disease.

## Introduction

**Figure 1:**
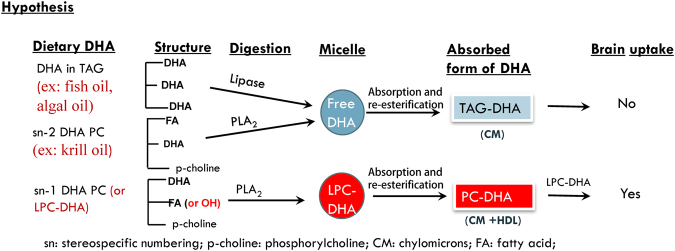
Proposed hypothesis. When dietary DHA is present in TAG (for example, fish oil) or in sn-2 position of PC (for example, krill oil), it is released as free fatty acid and absorbed as TAG in the chylomicrons. On the other hand, if DHA is provided in sn-1 position of PC, or as LPC, it will be absorbed as PC in chylomicrons and HDL. The PC-DHA is more likely to be transported into brain after conversion to LPC, compared to TAG-DHA, since the brain preferentially takes up DHA as LPC.

Docosahexaenoic acid (DHA), an essential omega 3 fatty acid, is uniquely concentrated in the brain, nervous tissues and retina, and is essential for the normal neurological development and function. The deficiency of DHA is associated with several neurological disorders, including Alzheimer’s, Parkinson’s, schizophrenia, and depression^1–5^. Unlike liver, the brain cannot efficiently convert dietary alpha linolenic acid (18:3, n-3) to DHA^6, 7^, and is almost completely dependent upon the uptake of preformed DHA from the plasma. However, dietary supplementation with the currently available preparations of DHA such as fish or krill oil^8^, algal DHA^9^, DHA-enriched egg phospholipids^10^ ethyl esters^11^ and sardines^12^ does not appreciably increase brain DHA levels in adult mammals, although peripheral tissues are enriched with DHA under the same conditions. One possible explanation for this is that DHA from the above supplements is hydrolyzed to free DHA by the pancreatic enzymes and absorbed as triacylglycerol (TAG) in chylomicrons (Fig. 1), whereas the brain uniquely takes up DHA in the form of lysophosphatidylcholine (LPC)^13–15^. The recent demonstration of a transporter at the blood brain barrier (Mfsd2a), which specifically transports LPC-DHA, but not free DHA^16^, further supports this mechanism. It is therefore necessary to increase the levels of LPC-DHA in plasma for an efficient enrichment of brain DHA. We propose that if dietary DHA is provided in the sn-1 position of phosphatidylcholine (PC) or in the form of LPC in the diet, it should escape the hydrolysis by pancreatic PLA_2_, and will be absorbed as PC-DHA (Fig. 1). The PC-DHA is more likely to be taken up by the brain after conversion to LPC-DHA in plasma or liver by the phospholipases, compared to TAG-DHA, which requires extensive metabolic transformations in the liver in order to form LPC-DHA. We have recently demonstrated in lymph fistula rats, that the amount of DHA that is absorbed in the form of phospholipid can indeed be increased by up to 5-fold by providing the DHA in the form of LPC, relative to free DHA, a surrogate for the currently used dietary supplements^17^. We also found that the incorporation of DHA into intestine-derived HDL is increased 2- fold during the absorption of LPC-DHA, compared to the absorption of free DHA. In this study, we tested the hypothesis that increasing DHA absorption in the phospholipid form not only increases brain DHA levels but also improves cognition and memory in normal adult mice. We compared the incorporation of dietary free DHA and LPC-DHA into the brain and other tissues, following daily gavage of the compounds in a corn oil vehicle for 30 days. Since it has been suggested that the presence of DHA at sn-2 position of LPC is important for efficient brain uptake and accumulation^14^, we have also compared the absorption and brain uptake of the two positional isomers of LPC-DHA (sn-1 DHA LPC and sn-2 DHA LPC). The results show that the DHA content of most regions of the brain is more than doubled by feeding either sn-1 or sn-2 DHA LPC, but not by feeding free DHA, which however enriched other tissues. Furthermore, the mice treated with both LPC-DHA isomers showed a remarkable enhancement of spatial learning and memory in the Morris water maze test. These studies are the first to demonstrate a targeted enrichment of brain DHA through diet leading to a functional improvement in memory in normal adult mice. If confirmed in humans, these studies have the potential to lead to novel nutraceutical approaches for the prevention and treatment of neurological disorders associated with low cerebral DHA levels, such as Alzheimer’s, Parkinson’s, schizophrenia, and depression.

## Results

### Incorporation of dietary DHA into plasma and other tissues

**Figure 2:**
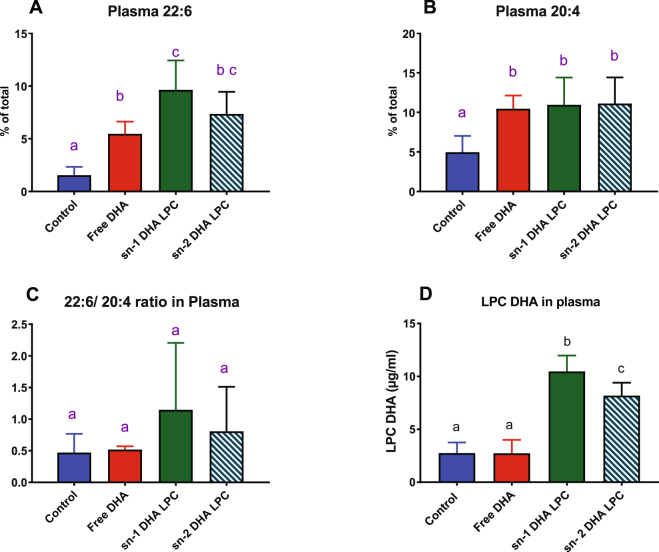
Effect of molecular carrier of dietary DHA on plasma levels (% of total fatty acids) of 22:6 (**A**), and 20:4 (**B**), on 22:6/20:4 ratio (**C**), and on LPC-DHA (µg/ml) (**D**). Results shown are mean ± SD (n = 8). Bars in each panel not sharing a common superscript are significantly different from each other (p < 0.05, one way ANOVA, and post-hoc Tukey test).

**Figure 3:**
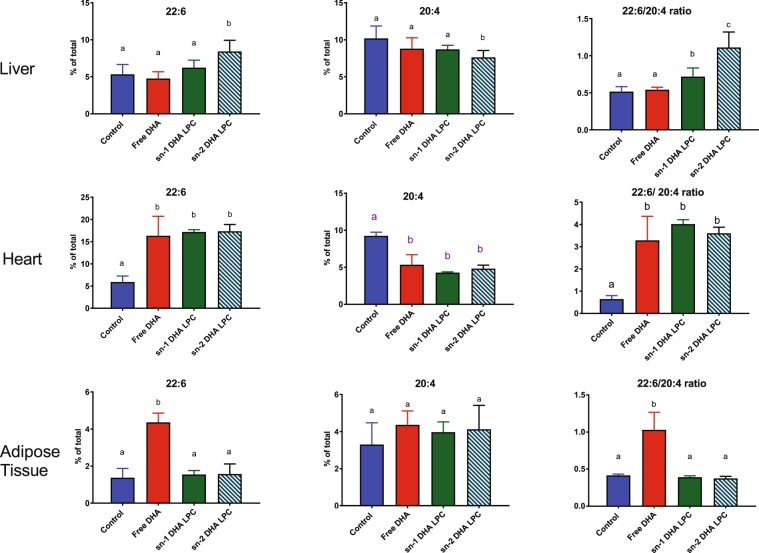
Effect of molecular carrier of dietary DHA on the concentrations of 22:6 and 20:4, and on 22:6/20:4 ratio in liver, heart and adipose tissue. The values shown are mean ± SD (n = 8). Bars in each panel without common superscripts are significantly different from each other (p < 0.05, one way ANOVA, followed by post-hoc Tukey test).

Wild type C57BL/6 mice, maintained on normal rodent chow, were gavaged daily with 80 µl of corn oil alone (control), or the corn oil containing 1 mg DHA in the form of free fatty acid, sn-1 DHA LPC, or sn-2 DHA LPC for 30 days. The food intake, and the body weights were not significantly different between the 4 groups of mice. Following the cognitive tests (see below), the mice were sacrificed and the fatty acid composition of various tissues was analyzed by GC/MS. The effect of dietary supplementation with different DHA preparations on the DHA content (% of total fatty acids) of plasma, liver, heart, and adipose tissue is shown in Figs 2 and 3. In addition to DHA, the percentages of arachidonic acid (ARA, 20:4) are shown, since DHA has been reported to replace predominantly ARA in most tissues, and since the 22:6/20:4 ratio provides one measure of the anti-inflammatory potential in the tissues^18^. In the plasma, there was a significant increase in the percentage of DHA by all three preparations of DHA, although the increase was greater in the groups treated with LPC DHA than the mice treated with free DHA (Fig. 2). However, there was also an increase in the percentage of 20:4 with all three DHA preparations, resulting in no significant change in the ratio of 22:6/20:4. The reason for the increase in plasma ARA is not clear, although it is decreased in most tissues where DHA is increased (see below). One possible explanation is that the tissue ARA replaced by DHA is released as free ARA into the plasma. The concentration of LPC DHA in plasma was also determined by LC/MS/MS. As shown in Fig. 2, the LPC DHA concentration of plasma increased significantly in mice treated with either isomer of LPC DHA, but not in mice treated with free DHA. Although we did not analyze the isomer composition of plasma LPC DHA because of insufficient material, the increase was 28% higher after the feeding of sn-1 DHA LPC compared to sn-2 DHA LPC.

The molecular species of DHA-containing lipids (PC, PE. TAG) in plasma were analyzed by LC/MS/MS, to determine the metabolic fates of the absorbed DHA. Most of the DHA-PC species were increased by treatment with both the LPC isomers, but not with free DHA (Fig. S1).There was no significant difference between the two LPC isomers, except for a greater increase in 18:1–22:6 PC in the sn-1 DHA LPC group. The molecular species of DHA-PE were also increased by the two LPC DHAs, but not by free DHA (Fig. S2). In contrast to the plasma phospholipids, the plasma TAG species containing DHA were increased more by free DHA than by the LPC DHA (Fig. S3).

The DHA concentration in liver was significantly increased only in the mice fed sn-2 DHA LPC, with a parallel decrease in the concentration of 20:4 and an increase in the 22:6/20:4 ratio (Fig. 3). Although the increase in DHA after feeding sn-1 DHA LPC did not reach statistical significance, the ratio of 22:6/20:4 was significantly increased. All three preparations of DHA increased the percentage of DHA in the heart, at the expense of 20:4, resulting in a significant increase in the ratio of 22:6/20:4 in all three groups of animals, compared to the controls. In contrast to the liver and heart, the adipose tissue showed an increase in DHA concentration, as well as in the 22:6/20:4 ratio after the feeding of free DHA, but not after feeding of either isomer of LPC-DHA. These results show that the dietary free DHA is predominantly directed to adipose tissue and heart in the form of TAG, whereas dietary LPC-DHA was directed to the brain in the form of phospholipid (see below).

### Incorporation of DHA into various regions of the brain

**Figure 4:**
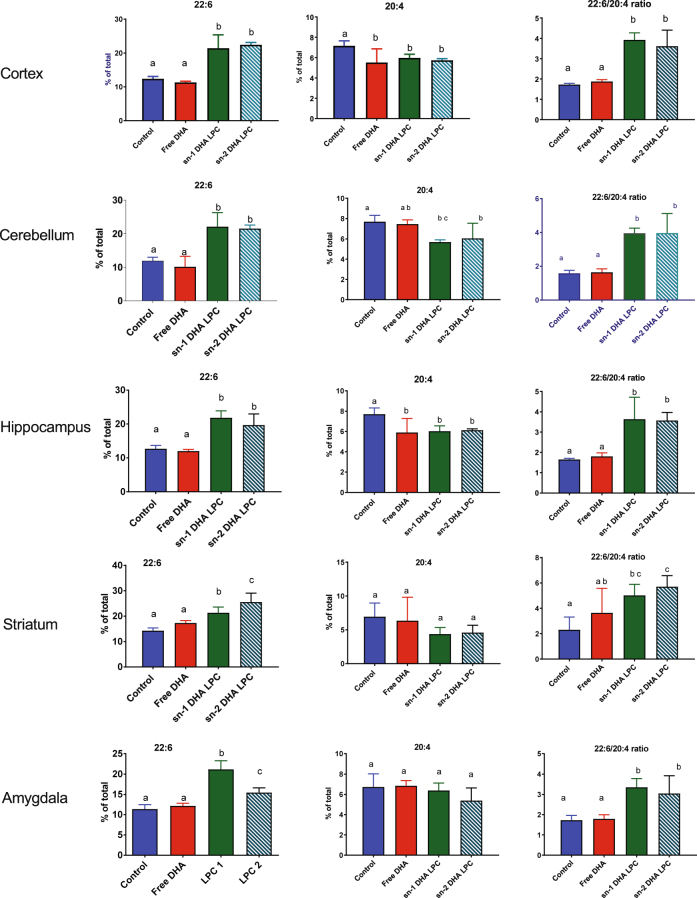
Effect of dietary treatment with free DHA, sn-1 DHA LPC, and sn-2 DHA LPC on the levels of 22:6 and 20:4 and on the 22:6/20:4 ratios in various regions of the brain. The values shown are mean ± SD (n = 8, for all regions, except for Amygdala, where n = 6). The bars in each figure not sharing common letter are significantly different from each other (p < 0.05, one way ANOVA, and post-hoc Tukey test).

The DHA and ARA concentrations of five brain regions are shown in Fig. 4. In contrast to the other tissues, all the brain regions were significantly enriched with DHA after the feeding either sn-1 DHA LPC or sn-2 DHA LPC, but not free DHA. The concentration of ARA was correspondingly decreased after feeding either isomer of LPC, and the 22:6/20:4 ratio more than doubled in all regions of brain. On the other hand, there was no significant increase in this ratio in any brain region after the feeding free DHA. These results show that the brain DHA responds specifically to treatment with dietary LPC-DHA. However, we found no difference between the two isomers of LPC-DHA in their ability to enrich brain DHA, although previous studies suggested that the sn-2 DHA LPC may be preferentially taken up by the brain^14, 15^.

**Figure 5:**
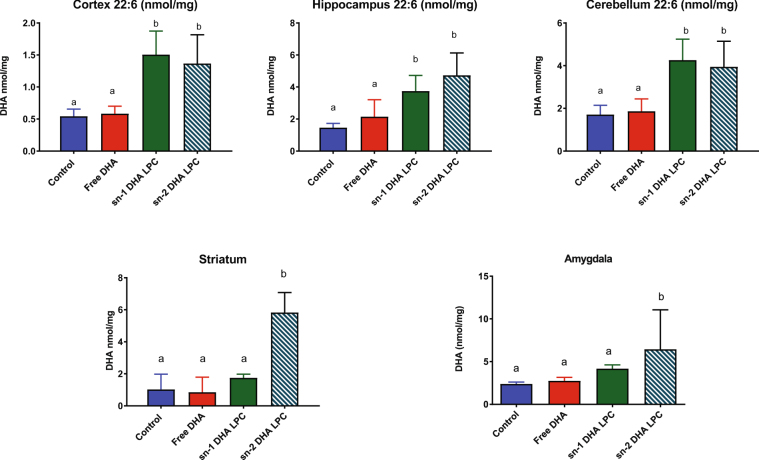
Effect of dietary free DHA, sn-1 DHA LPC, and sn-2 DHA LPC on the DHA concentration in various regions of brain, expressed as nmol/mg tissue. The values shown are n = 8, for all regions, except for Amygdala, where n = 6. Bars with non-identical letters on top are significantly different from each other in each panel (p < 0.05, one way ANOVA, and post-hoc Tukey test).

The increase in brain DHA content by dietary LPC-DHA, but not free DHA, was also evident when the results are expressed as nmol of DHA per mg wet weight of the tissue. By this measure, the DHA content increased by 2–3 fold in most regions of the brain after LPC-DHA treatment, except in the case of striatum and amygdala where the increase in the sn-1 DHA group did not reach statistical significance (Fig. 5). We also determined the molecular species of DHA-containing PC and PE in the hippocampus by LC/MS/MS, to investigate the incorporation profiles of DHA derived from dietary free DHA and LPC-DHA (Fig. S4). The amount of LPC-DHA, as well as most species of DHA-PC were increased by both isomers of dietary LPC-DHA, but not by free DHA. One exception was 20:4–22:6 PC, which was increased by free DHA, but not by the two LPC-DHAs. The major species of DHA-PE were also increased by the dietary LPC-DHAs, but not by free DHA (Fig. S5). Interestingly, the net increase in the amount of hippocampal PE-DHA was 4-fold higher than the net increase in PC-DHA by both isomers of dietary LPC-DHA, showing that the majority of DHA derived from dietary LPC-DHA was ultimately incorporated into brain PE.

### Effect of dietary DHA on brain function

**Figure 6:**
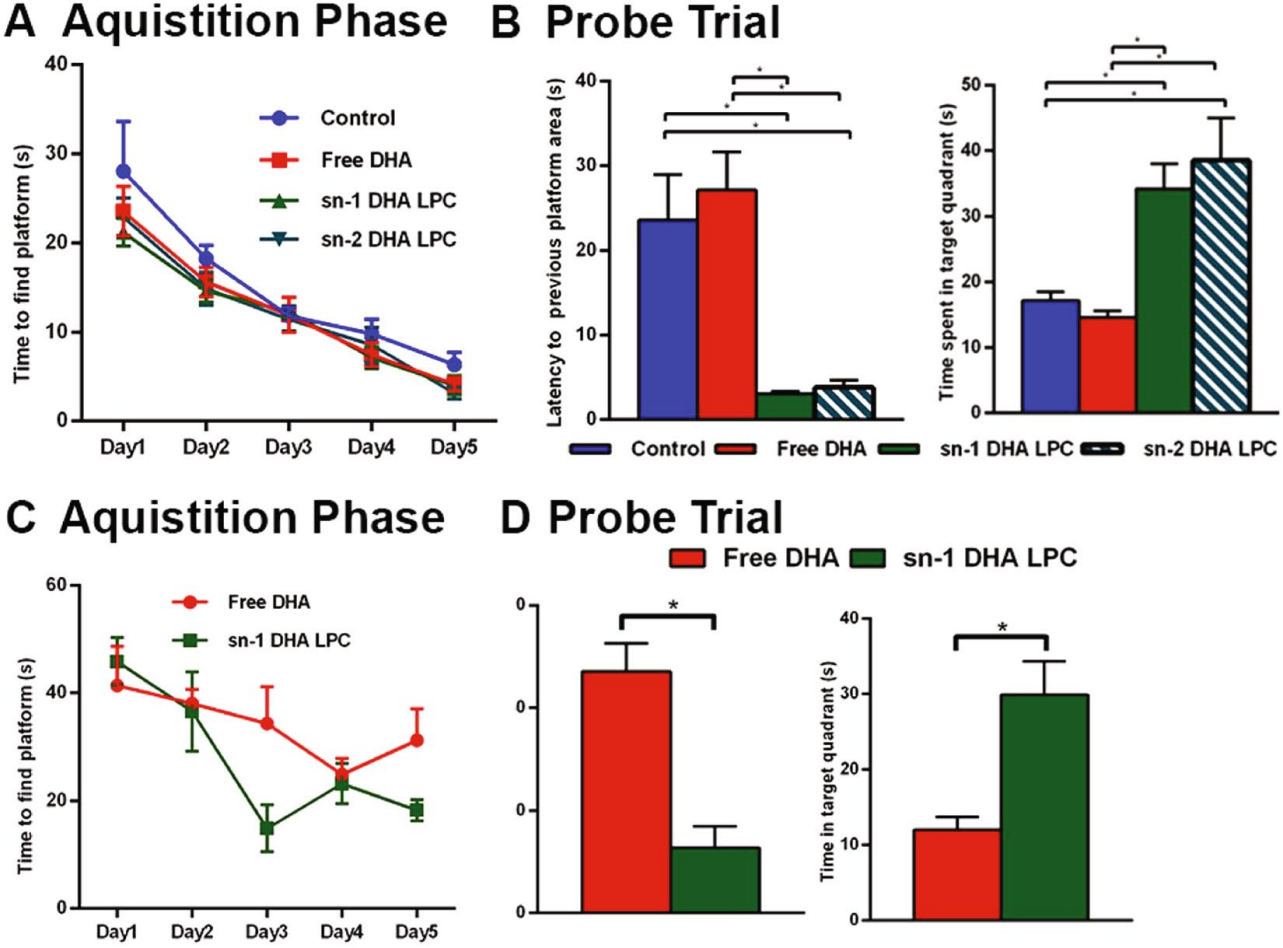
Improved memory in mice treated with LPC DHA compared to control or free DHA treated mice. In study 1 (**A** and **B**), Morris water maze tests of mice treated with vehicle (control), free DHA, sn-1 DHA LPC, and sn-2 DHA LPC for 1 month are shown. LPC DHA-treated mice did not differ from control or free DHA mice in the acquisition phase (**A**). In the probe trial (**B**) both sn-1 DHA LPC and sn-2 DHA treated mice reached the previous platform area with shorter latency time, and spent longer in the target quadrant. Data expressed as mean ± S.D (*n* = 8). *p < 0.05, by two-way ANOVA followed by Tukey’s post hoc comparisons. In a second study (**C** and **D**) to validate the above results, only 2 groups of mice were used and were treated either with free DHA or sn-1 DHA LPC for 1 month. As in the first study, sn-1 DHA LPC treated mice reached the previous platform area with a lower latency time, and spent longer in the target quadrant, compared to free DHA group (*n* = 5 each). Data are expressed as mean ± S.D. *p < 0.05 by two-way ANOVA followed by Tukey’s post hoc comparisons.

Although DHA has been reported to improve markers of cognition in DHA-deficient animals^19^ or in the disease states^20^, very few reports show a positive effects of dietary DHA on the cognitive functions of normal adult mice and rats. Even when the effects were demonstrated, the doses used were very high, in the range of 0.6 g to 2.4 g/kg body weight^20–23^, relative to what is practical in humans. Since we could more than double the brain DHA levels at very low amounts of LPC DHA (about 40 mg/kg body weight), we assessed whether LPC-DHA also modulated spatial learning and memory, as determined by the Morris water maze test. In the acquisition phase, all groups learned the location of the platform with no significant group differences (Fig. 6A). Notably, however in the probe trial (Fig. 6B), mice treated with either sn-1 DHA LPC or sn-2 DHA LPC located the previous platform area with a shorter latency time and spent longer time in the target quadrant compared to free DHA-treated or control mice (two-way ANOVA followed by Tukey’s post hoc comparisons). For example, both sn-1 and sn-2 DHA LPC treated mice found the previous platform area 7 times faster than the control and free DHA groups, and spent twice as long in the target quadrant.

To account for cohort effects and for validation, we conducted a second study in a smaller cohort of mice comparing only the free-DHA treated group with sn-1 DHA LPC treated group (Fig. 6C and D). Since we found no difference between vehicle control and free DHA groups in the above study, we did not include the vehicle control in the second study. Similarly, since there was no significant difference between the two isomers of LPC-DHA, we used only sn-1 DHA LPC. Although the data were more varied in the free-DHA group in this study, they were still consistent with the first study. In the acquisition phase, sn-1 DHA LPC mice learned the location of the platform faster by Day 5 (Day 1 vs days 2,3,4,5 and day 2 vs 5, two-way ANOVA followed by Tukey’s post hoc comparisons). However, the only significant difference for Free DHA mice was for day 1 vs 4 by two-way ANOVA followed by Fisher’s LSD test i.e. no multi-group comparisons. As for the first treatment study, in the probe trial sn-1 DHA LPC mice located the previous platform area with a lower latency time (free DHA- 47.1 seconds, sn-1 DHA LPC- 12.7 seconds) and spent longer time in the target quadrant compared to free DHA (free DHA- 12 seconds, sn-1 DHA LPC- 29.9 seconds).

In both MWM tests there were no group differences in average swim speed or total distance swam in the probe trial, and open field test showed that there was no significant change from the control mice in any of the treatment groups (not shown). Therefore, the beneficial effects of LPC DHA on memory were not related to general changes in locomotion.

### Effect of DHA on Brain derived neurotrophic factor (BDNF) levels

**Figure 7:**
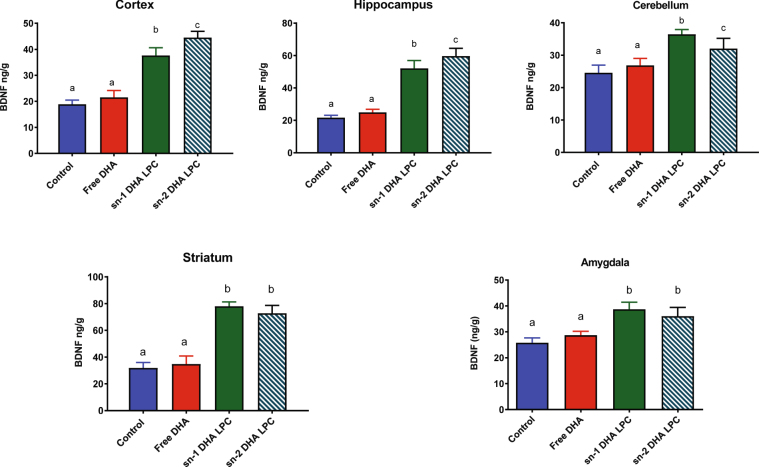
BDNF levels in the brain regions of mice treated with various molecular carriers of dietary DHA. Values shown are mean ± SD (n = 6). Bars with non-identical letters are significant from each other by one way ANOVA, and post-hoc Tukey test.

BDNF plays an important role in learning and memory, and is a potential downstream target of DHA^24–26^. Therefore, we determined BDNF concentration in the brain regions by ELISA to complement our MWM data. As shown in Fig. 7, while there was no change in BDNF content in any region of the brain after free DHA treatment, its levels were significantly increased by either sn-1 DHA or sn-2 DHA LPC. These results suggest that one of the mechanisms for the augmented memory in the mice treated with LPC-DHA is the increase in the BDNF levels.

## Discussion

Although it is well accepted that low brain DHA levels are associated with impaired brain function and memory, as well as several neurological disorders, attempts to increase DHA levels and improve brain function through the diet have been largely unsuccessful, especially in adult animals and humans^27–29^. We postulate that this failure is due to the presence of an intestinal barrier, which in conjunction with the blood brain barrier, prevents the DHA from the currently used supplements to enrich the brain. The proposed intestinal barrier is essentially the pancreatic enzymes, which release DHA from the dietary supplements in the form of free (unesterified) acid, which is then incorporated predominantly into chylomicron TAG (Fig. 1), whereas the blood brain barrier requires the DHA to be in lysophospholipid form for efficient enrichment of the brain^13–16^. In this study, we tested the hypothesis that if we can increase the absorption of DHA in the form of phospholipid, it is more likely to enrich the brain DHA and improve brain function. For this purpose, we compared dietary free DHA which is a surrogate for TAG-DHA (ex; fish oil, algal oil), as well as for the natural phospholipid-DHA (ex: krill oil), and which is absorbed as TAG, with dietary LPC-DHA, which is absorbed as PC^17^, with respect to the enrichment of brain DHA.

The results presented here show that the brain DHA levels can be increased by >2-fold in the normal adult mice by feeding either sn-1 DHA LPC or sn-2 DHA LPC, whereas treatment with an equal amount of free DHA did not appreciably increase the brain DHA levels. Although previous studies suggested that the sn-2 DHA LPC may be preferred over the sn-1 isomer for uptake by the brain^14, 15^ we did not find significant differences between the two isomers when administered orally. Furthermore, the increase in brain DHA by the two LPC isomers resulted in similar increases in BDNF levels, and a striking but similar improvement in spatial memory, as measured by Morris water maze test. One possible explanation for this is that the sn-2 DHA LPC is rapidly isomerized to the sn-1 isomer, and therefore the differences between the isomers is abolished. However, our recent studies show that unlike sn-2 16:0 LPC, the isomerization of sn-2 22:6 LPC is much slower even at physiological pH and temperature (Sugasini and Subbaiah, unpublished results). Therefore, the lack of differential uptake is not due to the isomerization of the sn-2 DHA LPC. It should also be pointed out that regardless of the LPC isomer used, the majority of the brain DHA was present in PE rather than in PC (Figs S4 and S5), suggesting that the DHA is released from LPC after its uptake, and reincorporated into membrane lipids of the brain.

The differential incorporation of free DHA and LPC DHA into the brain is not due to a difference in the amounts absorbed^17^, but apparently due to dissimilar metabolic fates after absorption. Thus the free DHA, which is absorbed predominantly as TAG in the chylomicrons, was incorporated significantly into adipose tissue and the heart, but did not accumulate in the brain. On the other hand, LPC DHA, which is absorbed in phospholipid form to a greater extent^17^, increased brain DHA levels, but had no effect on the adipose tissue DHA. In the liver, only the sn-2 DHA LPC increased the DHA concentration, whereas in the heart, all three preparations increased the DHA. It is known that chylomicrons, which have a very short half life in the circulation (<30 min), pass through the heart, adipose tissue and skeletal muscle before reaching the liver as remnants, and it is therefore likely that DHA from the absorbed TAG is largely picked up by these tissues. In fact previous studies showed that there is a preferential channeling of fatty acids derived from chylomicron TAG into adipose tissue^30^. In contrast, the DHA incorporated into phospholipids remains in the circulation much longer, because the chylomicron phospholipids are rapidly transferred to the HDL^31, 32^, which have a longer half-life of 12–24 h^33^. In addition, a significant fraction of dietary LPC-DHA is incorporated directly into intestinal HDL, mainly as phospholipids^17^. It should also be pointed out that the HDL phospholipids are the preferred substrates for three different enzymes in the plasma, namely lecithin-cholesterol acyltransferase^34^, hepatic lipase^35^, and endothelial lipase^36^, all of which are known to generate LPC-DHA, the preferred carrier of DHA into brain^13–15^.

The nature of the molecular carrier of DHA into the brain has been a matter of contention. Thus whereas Lagarde and co-workers^13–15^ reported that LPC-DHA is the preferred carrier of DHA through the blood brain barrier, the recent kinetic studies of Chen *et al*.^37^ concluded that free DHA is the major source of brain DHA, and that LPC DHA is only a minor contributor. The latter authors further suggested that there is no net increase in the DHA content of adult brain from either free DHA or LPC DHA of plasma. However, our results clearly show a net increase in brain DHA by dietary LPC DHA in the adult mice. Furthermore, this increase is accompanied by a marked increase in spatial learning and memory. Thus, we found a 7-fold decrease in the latency to previous platform area, and 2-fold increase in the time spent in the target quadrant by the LPC DHA treatment, compared to the control or free DHA treated animals.

One possible reason for the improved brain function is the increased levels of BDNF, which is known to play a critical role in learning and memory^38^. We found a significant increase in the BDNF levels after LPC-DHA treatment but not after free DHA treatment, thus showing a positive correlation of biochemical measurements with physiologic changes. The exact mechanism by which increases in brain DHA through LPC DHA leads to increase in BDNF levels is not clear. It is unlikely to be a direct effect of intact LPC DHA, since most of the brain DHA was present in PC and PE and only a small percentage was in LPC (Figs S4 and S5). It is more likely that free DHA released from membrane phospholipids not only acts as a precursor for various bioactive docosanoids^39^, but also modulates BDNF transcription. It has been shown that DHA increases BDNF synthesis through activation of CREB (cAMP response element binding protein) phosphorylation^25, 26^. DHA is also proposed to increase the transcription of BDNF by inhibiting hypermethylation of the promoter region of BDNF gene, which represses BDNF transcription^25^. Another possible mechanism is that since BDNF levels are negatively correlated with oxidative stress^40^, and since DHA is known to induce anti-oxidant enzymes^41^, the BDNF increase is a consequence of the induction of anti-oxidant enzymes which decrease the oxidative stress. A collateral benefit of delivering DHA to the brain in the form of LPC is that for each molecule of DHA entering the brain, a molecule of choline, an essential component of acetylcholine and of membrane lipids, is also delivered through the specific high affinity Mfsd2a transporter^16^. It is also possible that the free choline released from LPC hydrolysis contributes to the increase in BDNF, since previous studies showed that choline up-regulates BDNF, and down regulates its receptor TrkB (tropomyosin receptor kinase B), in cultured rat cortical cells^42^. An alternative explanation for the higher BDNF levels is that the DHA enrichment results in increased intracellular storage of BDNF, due to its decreased release. Although we cannot rule out this possibility at present, the improvement in memory by LPC-DHA suggests an increase in functionally active BDNF.

A previous study, in which female rats were fed DHA-enriched egg LPC of unknown composition, showed that the pups derived from these animals had increased DHA levels in some brain regions, and exhibited improved operant learning ability^43^, indicating increased passage of DHA through the placenta. However, the brain DHA levels of the mothers were not measured in this study. The present studies demonstrated, for the first time, the feasibility of increasing the net concentration of DHA in the adult brain through diet. These results therefore have the potential to provide cognitive benefits for adult mammals due to the increases in brain DHA and BDNF. Although these results need to be confirmed in humans, the present observations could lead to novel nutraceutical approach for the prevention and treatment of neurological diseases such as Alzheimer’s, Parkinson’s, traumatic brain injury, and depression, all of which are known to be benefited by DHA enrichment of the brain.

## Materials and Methods

### Animals

Male C57BL/6 mice (age 16 weeks) were purchased from Jackson laboratories (Bar Harbor, Maine). The mice were acclimated for 1 week before starting the daily gavage, and were provided rodent chow (Teklad LM 485, Envigo, Indianapolis, IN) throughout the experiment, in addition to the daily gavage of corn oil containing the various DHA preparations.

### Chemicals

Free fatty acids (15:0, 17:0, 22:3, 22:6) were purchased from Nu-Chek Prep Inc (Elysian, MN). Synthetic phospholipids (16:0–22:6 PC, 17:0-17:0 PC, 17:0 LPC, and 17:0-17:0 PE) were purchased from Avanti Polar Lipids (Alabaster, AL). All solvents, which were of LC/MS grade, as well as Mucor lipase (Lipozyme) were obtained from Sigma/Aldrich (St. Louis, MO).

### Preparation of sn 2-DHA LPC and sn 1- DHA LPC

sn-2 DHA LPC was prepared by the hydrolysis of 16:0–22:6 PC with immobilized Mucor lipase (Lipozyme) by a modification of the published procedure^44^. Briefly, 200 mg of Lipozyme was added to 100 mg of 16:0–22:6 PC, dissolved in 2 ml of 95:5 (v/v) ethanol: water, and was incubated in the dark at 37 °C for 24 h under nitrogen, in a metabolic shaker. The reaction mixture was dried under nitrogen and extracted with 8 ml of ethanol: water: hexane (2:1:1, by v/v) mixture to remove the free fatty acid in the hexane layer. The lower layer was concentrated under nitrogen and extracted by the Bligh and Dyer procedure^45^. The preparation contained >95% of lipid phosphorus in LPC DHA and the rest in unhydrolyzed PC, as determined by TLC, and was used for feeding studies without further purification. The sn-2 DHA LPC was stored in chloroform: methanol (9:1) at −20 °C. Over 90% of the LPC remained as sn-2 isomer for at least 4 weeks under these conditions. The sn-1-DHA LPC was prepared by exposing the sn-2-DHA LPC DHA to ammonia vapors at room temperature for 48 h in the dark under nitrogen. More than 95% of the 2-acyl LPC DHA was converted to 1-acyl isomer under these conditions, as determined by LC/MS.

### Feeding studies

All protocols were approved by the UIC Institutional Animal Care and Use Committee, and all the procedures were performed in adherence to the Institutional guidelines and regulations. Male C57BL/6 mice (16 week old) from Jackson labs, weighing 19–22 g were housed in rooms with a 12 h light/dark cycle and controlled temperature (22 ± 2 °C). The mice were allowed free access to standard rodent chow and water throughout the study. After a 1 week acclimation, the mice were divided randomly into 4 groups of 8 animals each, and the diet was supplemented with a daily gavage of 80 µl corn oil alone (control), or the corn oil containing 1 mg DHA in the form of free (unesterified) DHA, sn-1 DHA LPC, or sn-2 DHA LPC. This supplement provided a daily dose of approximately 45 mg DHA/kg body weight. The rodent chow did not contain any DHA, but contained 17.4 mg 18:3 (n-3)/g. After 30 days of feeding (and following the cognitive tests, see below), mice were fasted overnight and were anesthetized with ketamine (90 mg/ml) and xylazine (10 mg/ml). Blood was drawn by cardiac puncture into heparinized syringe, and plasma was separated by centrifugation at 1500 × g for 15 min at 4 °C. The mice were then perfused transcardially with ice-cold 100 mM phosphate-buffered saline (PBS), pH 7.4, and the liver, heart, gonadal adipose tissue, and brain were harvested. The brain was dissected into five regions: cortex, cerebellum, hippocampus, striatum, and amygdala. All samples were flash frozen in liquid nitrogen, and stored at −80 °C, until analysis.

A second group of mice was studied to confirm the behavioral effects of DHA, comparing only the effects of free DHA and sn-1 DHA LPC. The treatment protocol for this cohort was exactly same as the first cohort, excepting that only 5 animals were used for each group.

### Lipid extraction and analysis

Total lipids were extracted from plasma and various tissues by a modification of published procedure^46^. The tissue (up to 200 mg) was homogenized at 4 °C in a glass homogenizer three times with 800 µl each of 50% methanol in water containing 0.01 N HCl. A mixture of internal standards of tri-15:0 TAG, di-17:0 PC, di-15:0 PE, and 17:0 LPC (10 µg each) was included in the methanolic HCl. Chloroform (2 ml) was added and the sample vortexed for 30 s, followed by 1 ml water and vortexing for 30 s. The sample was centrifuged, and the chloroform layer was transferred to another glass tube, and the lipids were concentrated under nitrogen and re-dissolved in chloroform before further analysis.

### Analysis of Fatty acids by GC/MS

The fatty acid composition of lipids in all tissues was analyzed by GC/MS after conversion to methyl esters^17^. Briefly, the lipids were evaporated under N_2_ and dissolved in 0.5 ml toluene containing 25 μg of 22:3 free fatty acid and 250 μg butylated hydroxytoluene. Methanolic HCl (0.3 ml of 8% HCl in methanol) was then added, and the reaction mixture was heated under nitrogen at 100 °C for 1 h. The acid was neutralized by adding 1.0 ml of 0.33 N NaOH, and the fatty acid methyl esters were extracted twice with 3 ml of hexane. The pooled hexane extracts were evaporated under nitrogen and re-dissolved in 30 µl of hexane and 1 µl was injected into GC/MS. The analysis was carried out using a Shimadzu QP2010SE GC/MS equipped with a Supelco Omegawax column (30 m × 0.25 mm × 0.25 μ) as described previously^17^. Total ion current in the range of 50–400 *m*/*z* was used to quantify the fatty acids, using 17:0 as the internal standard.

### LC/MS analysis of DHA molecular species of PC, PE and TAG

LC/MS analysis of molecular species was performed on an ABSciex 6500 QTRAP mass spectrometer coupled with Agilent 2600 UPLC system, as described previously^17^. Quantitation of DHA-containing molecular species PC, LPC, PE and TAG was performed from the relative intensities of the various species and corresponding internal standards (17:0-17:0 PC, 17:0 LPC, 15:0-15:0 PE, and 15:0-15:0-15:0 TAG respectively). The data processing was carried out using Analyst 1.6.2 (ABSciex, USA).

### Morris water maze (MWM)

MWM was conducted as described in ref. 47 with slight modifications. All behavior was tracked in real time by an overhead camera and the videos analyzed using the ANY-Maze software (Stoelting). A circular pool (diameter, 120 cm; Height, 50 cm) was filled with water containing non-toxic tempera paint (maintained at 25 °C) and divided into equal-sized quadrants. Extramaze cues were placed in the four corners for spatial orientation. In the acquisition phase, mice were trained for 5 days (60 second trial time, 4 trials each day with a 20 minute inter-trial interval) to locate the hidden platform (diameter, 10 cm). The entry quadrant varied but the platform location remained constant. Latency to find the platform (s) was measured. One hour after the final acquisition trial a single 60 second probe trial was conducted with the platform removed. The latency to the target area (the previous platform location), time spent in the target quadrant, average speed and distance traveled were calculated.

### Statistical analyses

Results are expressed as mean ± SD for each experimental group. The data were analyzed by one way ANOVA followed by a post hoc Tukey test to compare the various groups. In some cases a two-way ANOVA followed by Fisher’s LSD test was used. p-values less than 0.05 were considered as statistically significant. All statistical analysis was performed using GraphPad Prism 7.03 and SPSS statistical software package version 17.0.

### Data Availability

All data generated or analyzed during the current study are included in this published article (and its Supplementary Information files).

## Electronic supplementary material

Supplementary Figures and Legends
