# Supplementary material for: Dietary docosahexaenoic acid (DHA) as lysophosphatidylcholine, but not as free acid, enriches brain DHA and improves memory in adult mice
Source: Sci Rep. 2017 Sep 12;7:11263. doi: 10.1038/s41598-017-11766-0 (PMC5596017; doi:10.1038/s41598-017-11766-0)
Supplement: Supplementary file 1 — Supplementary Figures and Legends [file 41598_2017_11766_MOESM1_ESM.pdf]

Supplementary Information

Dietary docosahexaenoic acid (DHA) as lysophosphatidylcholine, but not as free acid, enriches brain DHA and improves memory in adult mice

Dhavamani Sugasini , Riya Thomas, Poorna CR Yalagala, Leon M Tai, and Papasani V Subbaiah*

*Corresponding author

Supplement Fig S1

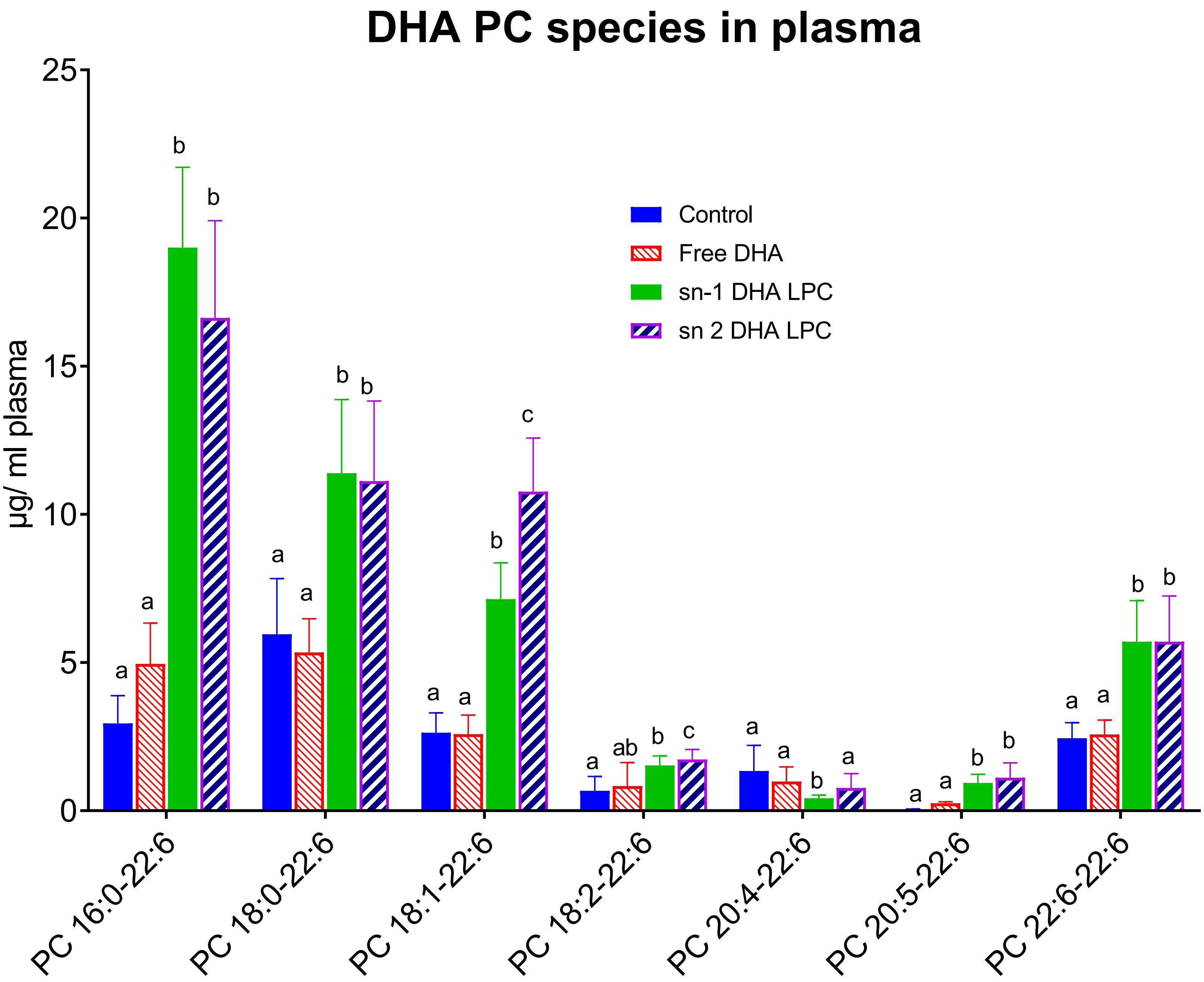

Supplement Fig S2

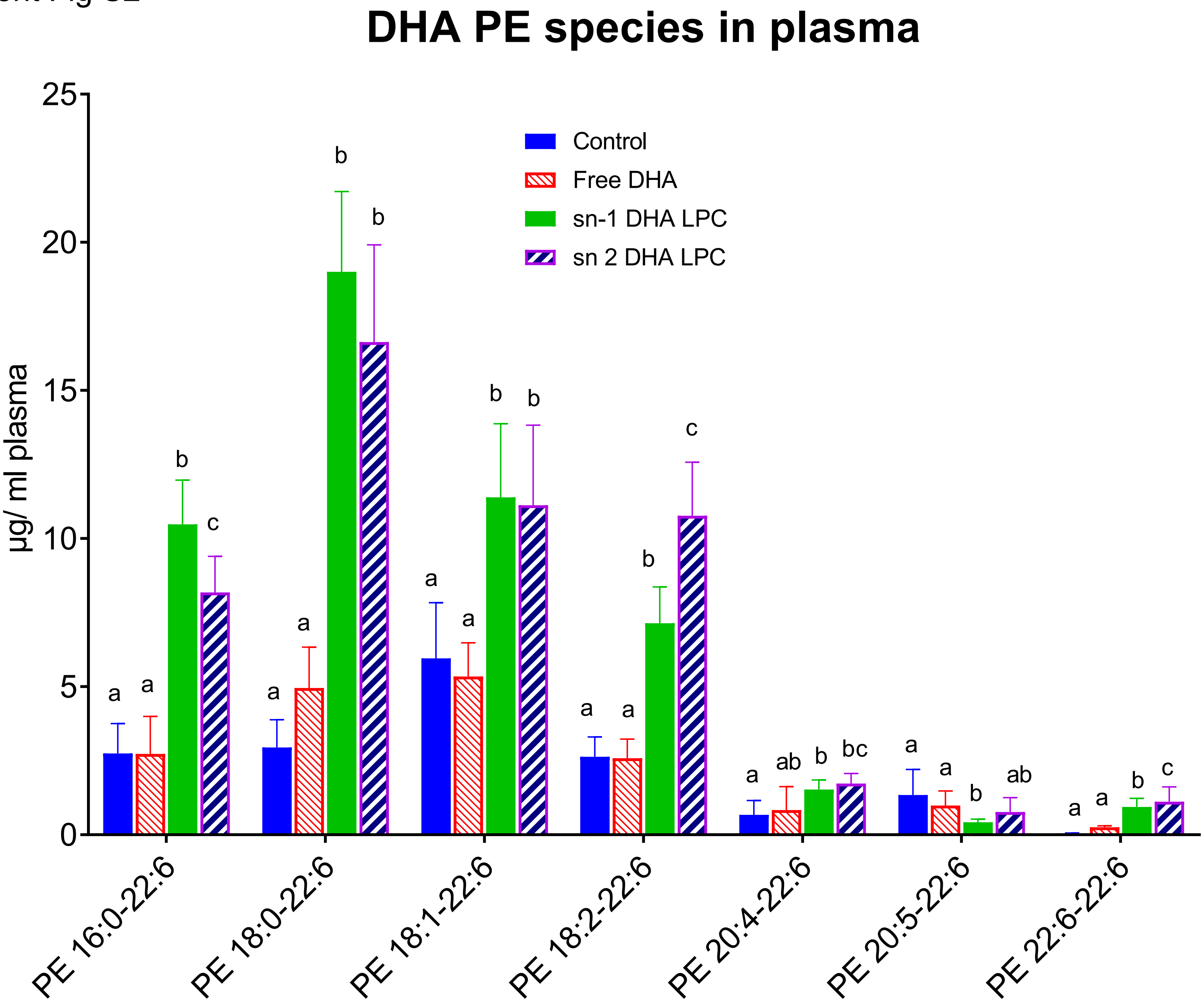

Supplement Fig S3

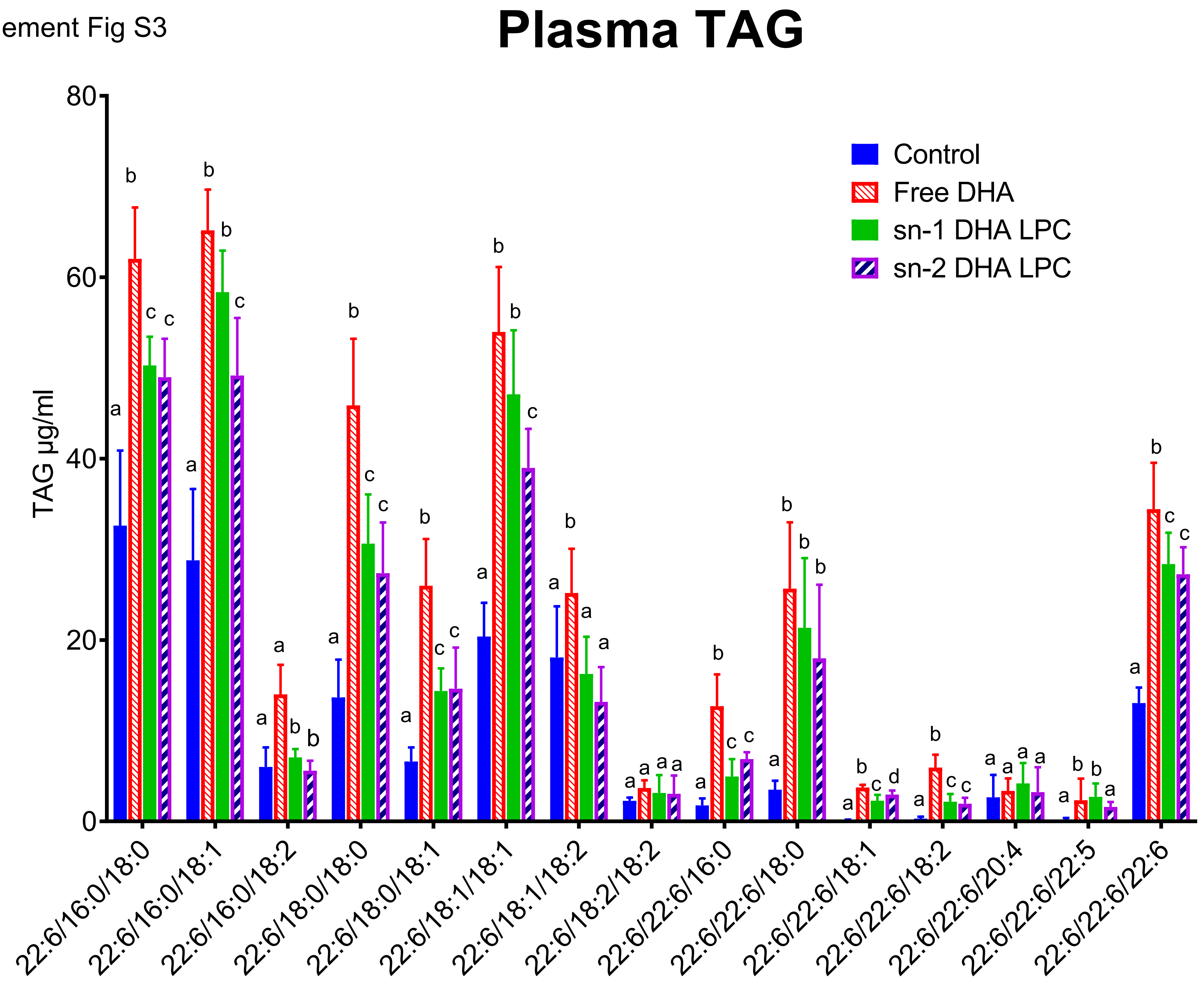

Supplement Fig S4

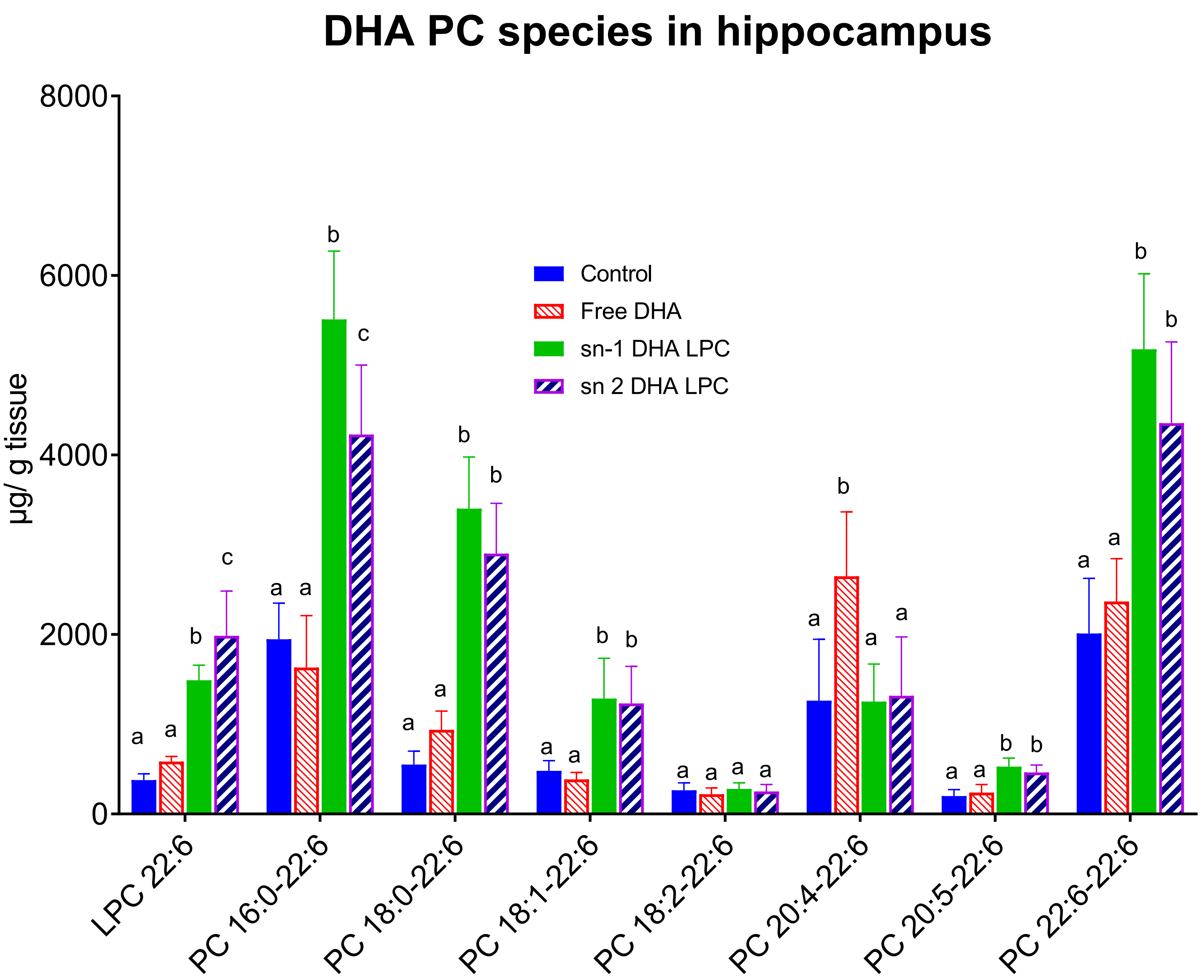

Supplement Fig S5

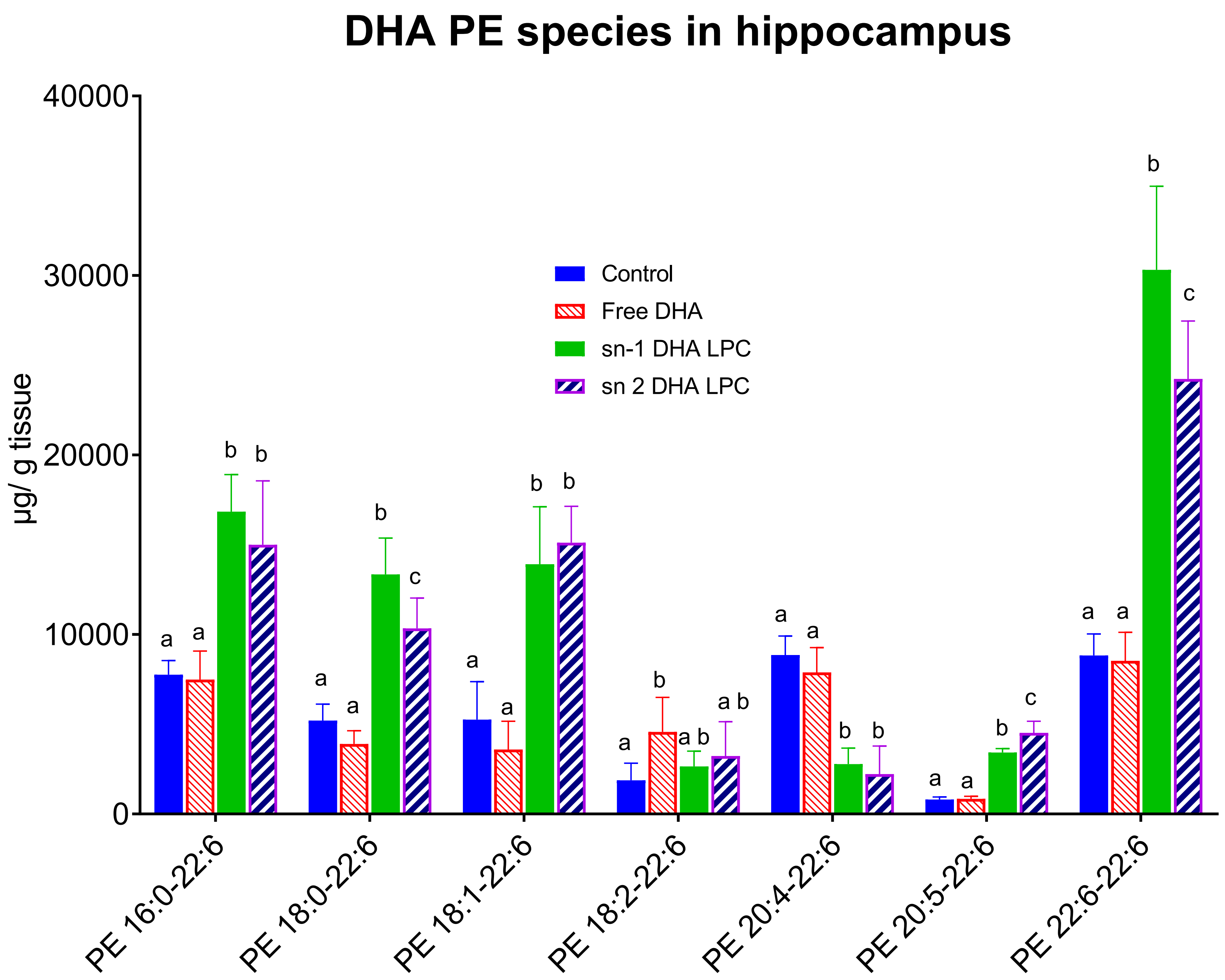

Legends for Supplementary Figures

Fig. S1. Molecular species of PC containing DHA in plasma of mice treated with various molecular carriers of dietary DHA. The PC species were determined by LC/MS/MS in MRM mode, using 17:0-17:0 PC as internal standard. Bars containing non-identical letters for each PC species are significantly from each other by one way ANOVA and post-hoc Tukey test.

Fig. S2. Molecular species of DHA-containing PE in plasma of mice treated with different molecular carriers of dietary DHA. The PE species were determined by LC/MS/MS in MRM mode, using 15:0-15:0 PE as internal standard. Values shown are mean ± SD (n=8), and the bars for each PE species not sharing common letters are significantly different from each other by one way ANOVA and post-hoc Tukey test.

Fig. S3. Molecular species of DHA-containing TAG in plasma after treatment with different molecular carriers of dietary DHA. The TAG species were analyzed by LC/MS/MS in MRM mode, using 15:0-15:0-15:0 as internal standard. Values shown are mean ± SD (n=8), and the bars for each TAG species not sharing common letters are significantly different from each other by one way ANOVA and post-hoc Tukey test.

Fig. S4. Molecular species of DHA-containing PC species in hippocampus of mice treated with different molecular carriers of dietary DHA. Values shown are mean ± SD (n=8), and the bars for each PC species without common letters on top are significantly different from each other by one way ANOVA and post-hoc Tukey test.

Fig. S5. Molecular species of DHA-containing PE species in hippocampus of mice treated with different molecular carriers of dietary DHA. Values shown are mean ± SD (n=8), and the bars for each PE species not sharing common superscript are significantly different from each other by one way ANOVA and post-hoc Tukey test.
